# Multiple Functions of the RNA-Binding Protein HuR in Cancer Progression, Treatment Responses and Prognosis

**DOI:** 10.3390/ijms140510015

**Published:** 2013-05-10

**Authors:** Jun Wang, Yan Guo, Huili Chu, Yaping Guan, Jingwang Bi, Baocheng Wang

**Affiliations:** 1 Department of Oncology, General Hospital, Jinan Command of the People’s Liberation Army, Jinan 250031, China; E-Mails: chuhuilicc@163.com (H.C.); guanyaping123@163.com (Y.G.); jingwangbi@hotmail.com (J.B.); baochengwang@hotmail.com (B.W.); 2 Department of Outpatient, Military Command of Shandong Province, Jinan 250013, China; E-Mail: wjun_2000_2000@126.com

**Keywords:** HuR, mRNA stability, progression, prognosis

## Abstract

The human embryonic lethal abnormal vision-like protein, HuR, is a member of the Hu family of RNA-binding proteins. Over the past decade, this ubiquitously expressed protein has been extensively investigated in cancer research because it is involved in the regulation of mRNA stability and translation in many cell types. HuR activity and function is associated with its subcellular distribution, transcriptional regulation, translational and post-translational modifications. HuR regulation of target mRNAs is based on the interaction between the three specific domains of HuR protein and one or several U- or AU-rich elements (AREs) in the untranslated region of target mRNAs. A number of cancer-related transcripts containing AREs, including mRNAs for proto-oncogenes, cytokines, growth factors, and invasion factors, have been characterized as HuR targets. It has been proposed that HuR has a central tumorigenic activity by enabling multiple cancer phenotypes. In this review, we comprehensively survey the existing evidence with regard to the diverse functions of HuR in caner development and progression. The current data also suggest that HuR might be a novel and promising therapeutic target and a marker for treatment response and prognostic evaluation.

## 1. Introduction

HuR is a member of the embryonic lethal abnormal vision (ELAV) family of RNA-binding proteins (RBPs) originally identified in *Drosophila* as essential for neural development [1]. It is the product of the human *ELAVL1* gene located on human chromosome 19p13.2 and was first cloned in 1996 [2,3]. The HuR protein is extensively expressed in many cell types, including adipose, intestine, spleen, and testis [4]. By contrast, other ELAV family members in mammals including HuB, HuC and HuD are almost exclusively found in neuronal tissues [4]. The expression of HuR and other RBPs are perturbed in several pathological conditions including human cancer, such as breast cancer, lung cancer, mesothelioma, ovarian cancer and colon cancer [5–7].

**Figure 1:**
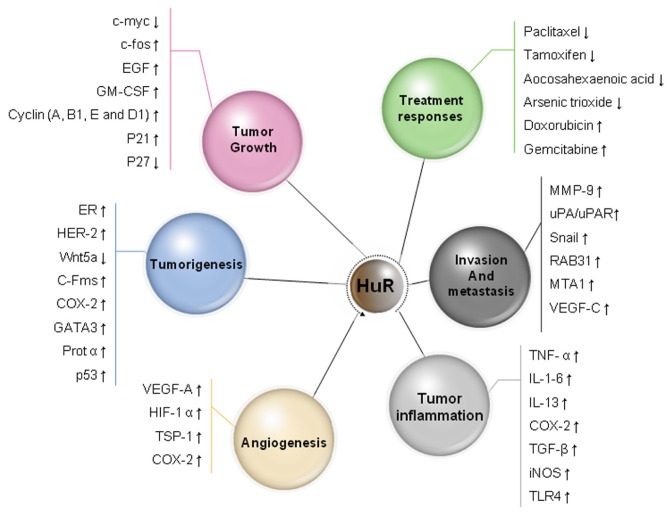
The diverse functions of HuR in cancer development and progression through the regulation of the stability or translation of target mRNAs that encode multiple cancer-related proteins. EGF, epidermal growth factor; GM-CSF, granulocyte-macrophage colony-stimulating factor; ER, estrogen receptor; COX-2, cyclooxygenase-2; GATA3, Trans-acting T-cell-specific transcription factor; ProTα, prothymosin α; VEGF, vascular endothelial growth factor; TSP1, thrombospondin 1; MMP-9, matrix metalloproteinase-9; uPA, urokinase-type plasminogen activator; uPAR, urokinase-type plasminogenactivator receptor; IL-6, interleukin-6; TNF-α, Tumor necrosis factor-α; IL-13, interleukin-13; TGF-β, transforming growth factor-β; iNOS, inducible NO synthase; TLR-4, toll-like receptor-4.

HuR has been reported to regulate the expression of many molecules by different post-transcriptional mechanisms, which are important components of eukaryotic gene expression, including mRNA trafficking, mRNA decay, and protein translation (Figure 1). Increasing evidence supports HuR is the first RBP that is shown to play a critical role in carcinogenesis and cancer progression by functioning as either an oncogene or a tumor suppressor regulating the expression of various target genes. Clinical data suggest that HuR overexpression is significantly related to specific clinicopathological features, advanced stage, positive lymph nodes, and poor survival in cancer patients. This review summarizes the recent findings and associations between HuR and cancer, especially in cancer development, progression, treatment responses, and prognosis.

## 2. Post-Transcriptional Regulation of Gene Expression by HuR

The regulation of a large subset of target mRNAs and protein translation by HuR is dependent on the molecular structure of the HuR protein. HuR binds to the cis-acting regulatory elements found the 5′untranslated region (UTR) or 3′UTR of the unstable mRNAs. As a trans-acting factor, HuR protein can recognize and bind the adenylate/uridylate (AU)- and U-rich elements (AREs) in the UTR of mRNA or poly (A) tail through three classic RNA recognition motifs (RRMs) [8]. Because ARE-mediated rapid degradation of mRNA is an important mechanism of post-transcriptional gene regulation in mammalian cells [9], the direct interaction between the HuR protein and AREs confers post-transcriptional regulation of gene expression by increasing both mRNA stability and/or protein translation [10].

During the regulation of mRNA stability, several RBPs, including AU-rich element RNA-binding protein 1 (AUF1), butyrate response factor 1 (BRF1), tristetraprolin (TTP), and KH-type splicing regulatory protein (KSRP), promote ARE-mRNA decay through the recruitment of the ARE-bearing mRNA to sites of mRNA degradation [11]. Similar to HuR, recently identified heterogeneous nuclear ribonucleoproteins (hnRNPs) are another family of RBPs [12]. HnRNP A1, hnRNP B1 and hnRNP K were aberrantly expressed in human cancer. Cytoplamic localization of hnRNPs were reported as effectors regulating cancer invasion and patient outcome [13–16], and interacted with HuR in heat-induced cells [17].

Recent studies linked the interactions between HuR and microRNAs (miRNAs). Functional investigations show that HuR and miRNAs might have the same mRNA functional site [18]. Competitive miRNAs, including miR-122, miR-548c, miR-494, miR-16, and miR-331, antagonize the contribution of HuR to the stabilization of target mRNA. Whether the stability of mRNA is increased or decreased depends on the binding strength of HuR and particular miRNAs with target mRNAs [19–23]. Conversely, cooperative miRNAs, such as let-7, miR-19 and the RNA-induced silencing complex (RISC), leads to the downregulation of protein production [24–26].

## 3. Shuttling of HuR from the Nucleus into the Cytoplasm

**Figure 2:**
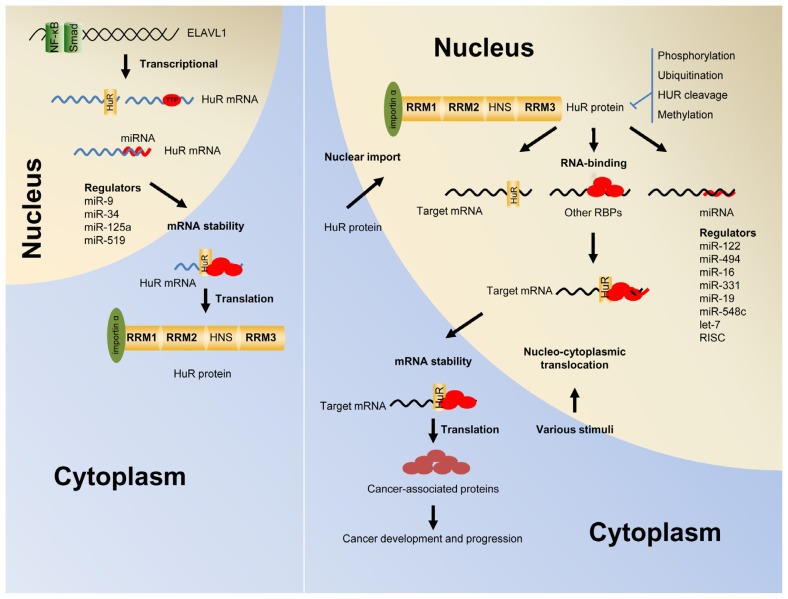
Autoregulation of HuR expression and function and its roles in regulating target mRNAs encoding cancer-related factors. Nuclear factor-κB and Smad control the HuR mRNA expression at the transcriptional level. HuR mRNA is also regulated by other RBPs (such as TTP and RNP C1), miRNAs and HuR protein itself, which influence HuR stabilization or protein translation. The nuclear import of HuR protein is associated with the activation of AMPK and elicits a dual modification of importin α1via acetylation on K22 and phosphorylation on S105. In response to various stimuli, HuR can be exported from the nucleus to the cytoplasm, where it stabilizes the target mRNAs. The nucleocytoplasmic shuttling mechanism of HuR is linked to HuR phosphorylation, ubiquitinylation, methylation and HuR cleavage, which effects the interaction of HuR with mRNA or/and its cytoplasmic accumulation. In addition, other RBPs and several miRNAs compete or cooperate with HuR, thereby their interaction affects the stability or translation of target mRNAs that encode proteins with multiple roles in cancer development and progression. NF-κB, nuclear factor-κB; TTP, tristetraprolin; miRNA, microRNA; RRMs, RNA recognition motifs; RBPs, RNA-binding proteins.

It is well known that intracellular HuR is predominantly localized within the nucleus of resting cells. Under various stimulations, HuR can bind to ARE-containing mRNAs in the nucleus. The HuR-mRNA complex is then transported to the cytoplasm. Once HuR is bound to the target transcript, it stabilizes the message and protects it from rapid degradation by exonucleases. HuR releases itself from the mRNA and returns rapidly to the nucleus after completing the process of stabilizing mRNA (Figure 2). This translocation from the nucleus to the cytoplasm appears to be an important aspect of HuR stabilizing function. Many stress stimulators have been reported to induce HuR shuttling, including ultraviolet radiation (UVC), lipopolysaccharide (LPS), chemical compounds, alterations in the microenvironment, cytokines, viral infection and hormone treatment [27–58]. Most of these reported exogenous stimuli result in a significantly increased cytoplasmic accumulation of HuR protein (Table 1).

The mechanism by which HuR shuttles from the nucleus to the cytoplasm is incompletely understood. However, several possible signaling pathways and coordinated molecules are involved (Table 2 and Figure 2). First, a novel shuttling domain, termed the HNS, is located in the hinge region between its second and third RRM. This domain allows HuR to shuttle back and forth between the nucleus and the cytoplasm. Second, several transport machinery components including transportins and the chromosome region maintenance 1, affect the nucleocytoplasmic shuttling of HuR [59,60]. Third, a number of kinases including checkpoint kinase 2 (Chk2), cyclin-dependent kinase 1 (Cdk1), protein kinase C (PKC), and p38 mitogen-activated protein kinase (p38 MAPK) phosphorylate HuR at serine/threonine residues and alter its subcellular localization [61–65]. Methylation of the HuR hinge region by coactivator-associated arginine methyltransferase 1 (CARM1) [66], and ubiquitination of HuR by an unknown E3 ligase [67], affect the cytoplasmic accumulation of HuR protein. In addition, the phosphorylation and methylation of the HuR change the binding activity of HuR with target mRNA [61,62,64]. In contrast to cytoplasmic accumulation, nuclear import of HuR protein is associated with the activation of AMPK, which elicits a dual modification of importin α1via acetylation on K22 and phosphorylation on S105 [42,68,69].

## 4. The Autoregulation of HuR Function and Expression

Although HuR has a crucial role in the post-transcriptional regulation of many transcripts, the regulation of its own function and expression remains obscure. As shown in Table 2 and Figure 1, HuR is controlled by many molecules at multiple levels. First, transcriptional regulation is essential for controlling the expression of HuR mRNA. The activation of AKT increases the binding of p65/RelA to a putative nuclear factor (NF)-κB binding site in the HuR promoter. This binding simultaneously enhances the cytoplasmic import of HuR and increases the stability of HuR targeted transcripts [72]. Another well-characterized transcriptional regulator is Smad, which was reported to bind to a motif in the GC rich 5′UTR of HuR and increases HuR mRNA expression [73]. Additionally, HuR mRNA is directly regulated by TTP, RNP C1, Mdm2, pp32 and Hsf1 [85–90]. Interestingly, HuR can be regulated by other ELAV family members and HuR itself. This regulation affects the stability of HuR mRNA and protein by influencing the HuR-mRNA interaction, mRNA stability, or protein production [83,84,90]. TTP is also an important RNA-binding protein and a TTP-HuR imbalance results in increased cell invasiveness through upregulation of cancer invasion factors including uPA, MMP-1, and MMP-13 [91]. Caspase-initiated HuR cleavage can affect the integrity of HuR protein [74]. Recently identified miRNAs, such as miR-9, miR-34a, miR-16, miR-125a, miR-29a, miR-200c, and miR-519, play crucial roles in regulating HuR expression through interaction of miRNAs with specific sites in the 3′UTR and 5′UTR of the HuR mRNA. The miRNAs lead to reduced expression of HuR mRNA and protein or alter the association of HuR with target mRNAs [74–82]. Interestingly, HuR can autoregulate its function and HuR can recognize and stabilize a long polyadenylation variant of HuR mRNA containing an ARE [83,90], or affect the cytoplasmic shuttling of HuR mRNA [92]. In contrast to TTP, genetic alterations of the *ELAVL1* gene do not routinely occur in tumor cells or primary tumors [72,93].

## 5. HuR Expression in Cancer

The HuR protein is encoded by the *ELAVL1* gene located on chromosome 19p13.2, which is a region correlated with various translocations and oncogenic mutations including T cell receptor gene [94], dynamin 2 [95], and intercellular adhesion molecules [96]. This gene was originally identified and cloned in 1996 [2]. Consistent with its function as an mRNA stability protein, high levels of cytoplasmic HuR have been found in oral, colorectal, gastric, lung, breast, ovarian, renal, skin carcinoma, and mesothelioma [97–109]. These studies revealed the association of HuR with cancer using immunohistochemical, RT-PCR or western blotting analysis. Clinical analyses showed that breast cancer cells with cytoplasmic HuR expression were usually associated with larger tumor size, estrogen receptor negativity, progesterone receptor negativity, p53 positivity and high tumor grade [110,111]. HuR was also associated with tumor stage in uterine cervical carcinoma [112] and with high tumor grade and poor differentiation in non-small cell lung carcinoma [102].

In cell culture studies, HuR expression is predominantly located in the nucleus of cancer cells and only small amounts of HuR are present in the cytoplasm. The immunohistochemical analyses for HuR localization show that HuR staining can be cytoplasmic, nuclear, or nuclear and cytoplasmic. Medium-to-strong HuR expression occurs in the nucleus of cancer cells as well as stromal cells adjacent to tumor, including macrophages and fibroblast cells. A weak or medium expression level of HuR is also found in the cytoplasm of cancer cells [97]. By contrast, there are few cells with a positive expression of cytoplasmic HuR or a lack of cytoplasmic HuR accumulation in normal tissues. Stromal cells and adjacent non-neoplastic tissue do not show cytoplasmic expression of HuR [97].

## 6. HuR Expression in Pre-Malignant Lesions

The status of HuR expression in human malignancies is apparently correlated with its expression in normal tissues and pre-malignant lesions. Blaxall *et al.*, first detected elevated HuR expression in urethane-induced neoplasia and butylated hydroxytoluene-induced compensatory hyperplasia in mouse lung tissue [113]. Non-cancer, precancerous lesions and tumor tissues exhibit a distinctive HuR expression profile that may have practical implications [114,115]. A comparison of HPV-induced low-grade and high-grade pre-malignant lesions and cervical cancers showed the expression of all RBPs increased in neoplastic lesions. The highest RBPs expression occurred in cervical cancers [116] with a similar expression profile to proliferating cell nuclear antigen. These findings indicate the nucleocytoplasmic translocation and cytoplasmic presence of HuR is necessary for its activity and function in several types of carcinomas.

The cellular and subcellular localization of HuR may be a surrogate for HuR function in cancer development and progression. The mechanism underlying HuR mediated carcinogenesis and cancer development remains unclear. However, its mRNA stabilizing function is required for cancer development. In 2003, López de Silanes *et al*., found HuR-overexpressing RKO cells produced larger tumors than control cells. A reduction in HuR expression through RNA interference or antisense significantly slowed the growth of colon tumors in nude mouse xenografts [117]. The increased expression of HuR occurs in virtually all cancer tissues compared to the normal-tissue counterparts and collections of HuR-regulated mRNAs were identified in colon cancer cells by cDNA arrays [118].

An important carcinogenesis related factor is cyclooygenase-2 (COX-2). This protein is an inducible enzyme critically involved in the synthesis of prostaglandins. The prostaglandins have been widely studied because HuR regulates their abnormal expression, especially in gastric and colorectal carcinoma. These studies showed a statistically significant difference between early-onset gastric cancers and conventional gastric cancers based on COX-2 and HuR expression status [119]. This difference was similar when the expression of COX-2 and HuR were evaluated in normal epithelium, high-grade prostatic intraepithelial neoplasia and prostate carcinoma [114]. The increased HuR expression and cytoplasmic localization were present in 76% of adenomas and 94% of adenocarcinomas. Only low levels of HuR are present in normal colon tissues [120]. Additional studies also supported this conclusion in other cancer types [114,121]. The competition between HuR and TTP for binding to COX-2 mRNA can lead to the deregulation of COX-2 during colon tumorigenesis [120]. Furthermore, HuR binds to many mRNAs and promotes their stabilization. The HuR target mRNAs include oncogenes, [76,122,123], cyclins [22,124,125], cyclin-dependent kinases, methyltransferases [126,127], inflammatory factors [32,128–130], and apoptosis-related molecules [20,131,132]. Additionally, HuR is also responsible for the tight regulation of tumor suppressors p21 and Wnt family protein Went-5a [133,134], indicating its role in tumor suppression. Thus, there is a growing body of evidence suggesting HuR-mediated post-transcriptional regulation of its target mRNAs is critical for neoplastic transformation and cancer development.

## 7. HuR Function in Tumor Angiogenesis

Tumor cells can promote vascular growth or angiogenesis through different mechanisms. Angiogenesis subsequently contributes to tumor growth and helps cancer cells enter the peripheral circulation. Vascular endothelial growth factor-A (VEGF-A), interleukin-8 (IL-8), hypoxia-inducible factor-α (HIF-α), and COX-2 have a predominant role in controlling this process [135]. There are several levels of regulation for these angiogenic factors including transcription, mRNA stability, and translation. However, post-transcriptional mechanisms are particularly involved in controlling the expression of these angiogenic factors. Many clinical investigations have shown a positive relationship between cytoplasmic HuR accumulation and VEGF-A [136,137], VEGF-C [101,109], COX-2 [103,104,109,138–140], and IL-8 [130] in human tumor samples, whereas cytoplasmic staining of HuR was not associated with VEGF-D expression in bladder cancer [109]. The association of VEGF-A with HuR has been previously reviewed by Yoo *et al*. [133]. Moreover, HuR was found to correlate with increased blood microvessel density [102,135,141]. Furthermore, cytoplasmic HuR was significantly associated with larger tumor size in various human malignancies [109–111,142].

The increased cytoplasmic HuR expression is responsible for upregulating mRNA and the protein expression of important molecules by interacting with the mRNAs in cancer cells responding to different types of stress [49–51,130]. In addition, HuR was associated with the upregulation of VEGF-A and COX-2 in tumor endothelial cells. This result suggests HuR plays a critical role in activating angiogenesis in the tumor endothelium [143]. Our previous study showed HuR was involved in IL-1β-induced COX-2 and VEGF-C expression. HuR levels positively correlated with increased lymphatic microvessel density, which indicates a role of HuR in tumor-associated lymphangiogenesis [101,102]. Interestingly, in triple negative breast cancer, HuR overexpression significantly interfered with tumor growth, which conflicts with other reports showing the pro-growth function of HuR. The putative mechanism of this finding is that HuR had an anti-angiogenetic effect in orthotopic mouse models. HuR increased the expression of TSP1and but downregulated VEGF-A that are normally increased by HuR [144]. Furthermore, HuR can differentially regulate unique subsets of mRNAs in estrogen receptor negative and estrogen receptor positive breast tumors [145]. Its interaction with miRNAs affects the distribution or targeting of HuR to specific mRNA [24]. As a result, tumors with a different aggressive phenotype could have a specific expression pattern of RNA-binding proteins. All RNA-binding proteins should be analyzed before utilizing HuR as a potential therapeutic target in the future.

## 8. HuR Function in Cancer Invasion and Metastasis

Tumor cells have the ability to invade adjacent tissues or to enter the peripheral circulation and proliferate in distant organs, especially in lung, liver, bone and brain. In normal liver endothelial cells, HuR promoted gap junction mediated intercellular communication and adherens junction integrity by enhancing the expression of Cx43 and beta-catenin [146]. Clinical studies have demonstrated the cytoplasmic expression of HuR was associated with lymph node metastasis in non-small cell lung carcinoma [101], colon carcinoma [147], upper urinary tract urothelial carcinoma [148], and showed a correlation with advanced diseases [111]. Several recent reports indicated the cytoplasmic levels of HuR significantly were increased in tumors with lymphatic/vascular invasion compared to tumors without vessel invasion in cervical carcinoma, colon carcinoma, and ductal in situ carcinoma of the breast [109,110,147,149]. A high cytoplasmic-to-nuclear ratio was also significantly correlated with lymph node involvement at presentation [150].

HuR has been proposed to favor the process of cancer progression by regulating the expression of invasion and metastasis related genes. Studies have shown uPA and its receptor, which are well-known invasion factors, are tightly regulated by HuR mitogen-activated protein kinase-activated protein kinase 2 at the transcriptional level [151]. Another important HuR regulated factor is Snail, which is a hallmark of epithelial-mesenchymal transition and plays an important role in the invasion of mammary carcinomas [152]. In addition, matrix metalloproteinase-9 (MMP-9) was also found to be regulated by HuR. This was supported by data indicating HuR knockdown [125], kalopanaxsaponin A [47], dihydroavenanthramide [153], or radix clematidis extract [154] treatments significantly inhibited MMP-9 expression and HuR cytoplasmic translocation by different signaling pathways. HuR silencing in an immortalized breast epithelial cell line reduced anchorage-independent growth, cell invasion, and increased programmed cell death by targeting CTGF and RAB31 transcripts [149]. Thus, HuR-mediated cancer progression follows the upregulation of HuR-targeted mRNAs encoding extracellular proteases and proteins that alter the aggressive potential of cancer cells or change the extracellular matrix. Recently, Hsia *et al*., found that lapatinib-induced breast cancer invasiveness is caused by the downregulation of miRNA-7 and induction of epidermal growth factor receptor (EGFR) and COX-2 by a HuR-mediated posttranscriptional mechanism [155].

## 9. HuR and Drug Resistance and Sensitivity

Drug treatments are commonly used in the clinical management of cancer. The main clinical obstacle to successful solid tumor therapy is drug resistance. HuR has recently been implicated in inducing drug resistance. In breast cancer MCF-7 cells, the cytoplasmic accumulation of HuR was proposed as a key mediator in the development of tamoxifen resistance, due to its ability to stabilize specific transcripts that encode drug-resistant proteins and activate subsequent MAPK and JNK signaling [33]. In glioma, the activity of HuR is a contributing factor in the onset of drug resistance and tumor growth by increasing the expression of bcl-2 [156]. High Tubulin beta-3 chain (TUBB3) expression is related to a poor chemotherapy response and adverse prognosis in gastric carcinoma, pancreatic ductal adenocarcinoma and non-small cell lung carcinoma [157–159]. The results from Raspaglio *et al*., suggest that cytoplasmic HuR staining was also positive in tumors with high TUBB3 expression [160]. In A2780 ovarian cancer cells, the combination of HuR and miR-200c regulated the expression of TUBB3 and was linked to the abrogation of the resistant phenotype for both paclitaxel and cisplatin [75,160]. The introduction of a miR-34a precursor into paclitaxel and hormone-resistant prostate cancer cells caused a decrease of HuR, bcl-2, and SIRT1 expression and inhibition of the SIRT1 3′UTR activity. This result suggests HuR may be involved in paclitaxel resistance. Additionally, HuR bound to the transforming growth interacting factor mRNA 3′UTR and prevented it from degradation in response to arsenic trioxide in hepatocellular carcinoma, which suggests a connection between HuR function and arsenic trioxide resistance during anti-cancer therapy [161]. Recent study showed that a worse event-free survival rate in some triple-negative breast cancers was associated with over-expressed EGFR and increased COX-2 mRNA stabilization by HuR [155].

Cumulatively, these studies indicate a HuR-dependent mechanism for cancer cell survival and responses to chemotherapeutic or molecularly targeted drugs. HuR should be considered as a new therapeutic target to override drug resistance. For example, docosahexaenoic acid treatment sensitized nr-HaCaT cells to UVR-induced apoptosis by increasing the bax/bcl-2 ratio and caspase-3 activity while also reducing COX-2 levels. Furthermore, the transfection of nr-HaCaT cells with HuR siRNA can mimic the proapoptotic effect of docosahexaenoic acid by downregulating HuR expression [162].

HuR has also been implicated in mediating drug sensitivity. Costantino *et al*., found that HuR mediate gemcitabine efficacy by stabilizing the mRNA of a key gemcitabine metabolic enzyme, deoxycytidine kinase. The increased deoxycytidine kinase metabolizes and thereby activates gemcitabine by metabolizing the prodrug gemcitabine into its di- and tri-phosphate metabolites [35]. The cytoplasmic status of HuR correlates with worse pathologic features as assessed by T staging. Additionally, HuR status is a strong positive predictive marker for overall survival in patients treated with gemcitabine [142]. Latorre *et al*., investigated the role of the HuR protein during the cellular response to the anticancer drug doxorubicin. The results demonstrated *in vitro* selection of doxorubicin resistant MCF-7 cells overexpressing the multidrug resistance ABCG2 transporter had significantly downregulated HuR. The results were consistent with the downregulation of HuR targets and by loss of rottlerin toxicity [163]. HuR enhanced TOP2A translation and induces apoptosis by competing with miR-548c-3p and stabilizing TOP2A mRNA. The combined actions of HuR and miR-548c-3p control TOP2A expression levels and determine the effectiveness of doxorubicin and increase cell apoptosis [20]. In breast cancer, a synonymous polymorphism (rs3746083) of another RNA-binding protein tristetraprolin was significantly associated with a lack of Trastuzumab response in patients with HER2-positive-breast cancer [164].

Thus, HuR exerts various regulatory functions and controls the expression of different target mRNAs via post-transcriptional mechanisms. HuR regulates tumor responses to cytotoxic agents, small-molecule antagonists, and molecular targeted agents. Recently, HuR was found to exert a different role in regulating unique subsets of mRNAs in estrogen receptor negative and estrogen receptor positive breast cancer using RNA immunoprecipitation and microarray analysis [146]. We hypothesize that many therapy resistance and sensitivity genes are regulated by HuR and other RNA-binding proteins. The expression of HuR could be a new mechanism of chemotherapy responses in cancer. The role of HuR in drug responses can vary widely in different cells and tissue types or in different stages of cancer development and progression.

## 10. Prognostic Significance of HuR in Human Carcinoma

Usually, tumor-node-metastasis stage is the best prognostic index for operable cancer patients. However, each patient’s prognosis varies significantly within this staging system. Understanding the pathological and molecular factors that identify patient subsets suitable for aggressive systematic treatment is particularly important for early-stage cancer patients.

HuR is the firstly identified mRNA stability protein, expression of which has been linked to changed prognosis in cancer patients. The association between HuR expression and cancer patient survival is summarized in Table 3. In the majority of these published retrospective studies, immunohistochemistry was used to investigate the intercellular expression pattern of HuR in human malignancies, and the cytoplasmic expression of HuR was associated with poor survival, disease-free survival, metastasis-free survival, or overall survival, using univariate or multivariate analysis [34,103,105,111,112,143,165–167]. By quantitative immunohistochemistry, Laurlola *et al*., found the low ratio between nuclear and cytoplasm retained an sensitive prognostic significance relative to the risk of metastasis and death for patients with early stage lung adenocarcinoma [150]. However, studies in pancreatic carcinoma patients that received potentially curative pancreatic resection showed HuR cytoplasmic staining was a positive predictor for gemcitabine sensitivity and good prognosis [35,143]. Most of these studies investigating nuclear HuR status did not find a relationship between nuclear staining and prognosis. However, Yi *et al*., demonstrated HuR nuclear expression also correlated with reduced disease-free survival in ovarian carcinoma [168]. A study in prostate carcinoma patients concluded an opposite result [139]. In a set of 560 patients with colorectal adenocarcinom, tissue microarray analysis with a quantitative, automated immunofluorescent microscopy system indicated that the immunoreactivity for total HuR predicts poor prognosis [141]. Conversely, HuR was a good prognostic indicator for disease-free survival in breast cancer [169], when total cellular expression of HuR in cancer tissues were analyzed by western blotting. These results are consistent with an experimental investigation both *in vivo* and *in vitro* that showed HuR over-expression impaired tumor growth and reduced angiogenesis [144]. In other studies, the nuclear-to-cytoplasmic ratio has an influence on overall survival of patients with lung adenocarcinoma or colorectal carcinoma [141,149]. Interestingly, high levels of HuR mRNA correlated with longer overall survival in patients with stage I–IV breast cancer, but the results were not statistically significant [170]. Recently, over-expression of hnRNPs indicates a poor prognosis for patients with various human cancers [13–16], and genetic polymorphisms of TTP gene but not HuR gene polymorphisms were associated with poor prognosis of breast cancer patients [97].

In conclusion, HuR may exert a complex role in various types of human cancer. HuR protein level but not mRNA level may be very variable among cancer cells and tumor tissues. This expression pattern differs from the expression pattern of TTP. Both detection of HuR total protein and its cytoplasmic abundance may be useful in determining its prognostic value in different subsets of human malignancies. However, present data are based on retrospective studies regarding prognostic indicator is lower than provided by randomized controlled trials. The sample size of tumors studied in individual investigations varied. Further investigations are needed to reveal the prognostic value of HuR for patients with different stages or other malignant behaviors using a standard methodology for HuR detection in large prospective clinical trials.

## 11. Conclusions

A comprehensive investigation of the biological activity of HuR indicates it is a crucial regulator of post-transcriptional gene expression and has a central role in cancer [171]. Its multiple functions are linked to its ability to recognize, bind, and stabilize a large subset of ARE-containing mRNAs. The HuR target mRNAs encode a variety of factors required for cancer cell proliferation, survival, angiogenesis, invasion, and metastasis. Many HuR bound target mRNAs can be detected using cDNA array hybridization [172]. Recently, methods based on RNA-protein crosslinking, cross-linking and immunoprecipitation (CLIP), photoactivatable ribonucleoside-enhanced CLIP, and whole-transcript expression profiling, were developed to identify transcriptome-wide HuR binding sites [173–175]. These methods are helpful to elucidate regulatory mechanisms of HuR in mRNA processing and HuR-dependent antagonism of proximal miRNA-mediated repression. HuR expression and subcellular localization is aberrant in human tumor tissues. Unlike other RBPs, HuR mRNA levels change less dramatically in cancer than HuR protein. In response to various stimuli, HuR protein has the ability to move from the nucleus to the cytoplasm, where it stabilizes target mRNAs. Post-transcriptional modifications appear to control HuR abundance, localization, and binding to mRNAs. Therefore, inhibition of the cytoplasmic accumulation of HuR concomitantly with the administration of current therapeutics may lead to successful treatment strategies.

Establishing the molecular mechanism of HuR regulation could be useful in identifying new targets for drug design. These strategies may include direct inhibition of HuR expression using HuR interference and HuR antisense, inhibition of HuR translation, suppression of HuR translocation between the nucleus and cytoplasm and using exogenous modulators such as kinase inhibitors. Additionally, published studies showed the ARE-harboring mRNAs are differentially regulated through the concerted efforts of RBPs such as HuR, AUF1, TTP, BRF1, and KSRP with miRNA-mediated effects. The coordinated actions of HuR or other RBPs add a complexity to current understanding of regulatory mechanisms of gene expression in cancer development and progression. These results suggest targeting other RBPs or miRNAs can be developed as additional strategies for cancer treatment.

Recently, both natural and synthesized chemical compounds were found to affect HuR accumulation and attenuated the expression of cancer-related mRNAs. For example, suberoylanilide hydroxamic acid [39], inhibited cell transformation by suppressing HuR expression. Ginkgo biloba extract [71] inhibited cell proliferation by decreasing cytoplasmic levels of HuR. Green tea may regulate HuR expression at the transcriptional level and control inflammation and MMP-9 upregulation [45,70]. By contrast, kalopanaxsaponin A [47] and triptolide inhibited MMP-9 and COX-2 expression, respectively, by suppressing HuR cytoplasmic accumulation [48] (Table 1). In addition, exogenously and endogenously produced nitric oxide reduced the expression of HuR mRNA and protein and increased the degradation of MMP-9 mRNA [176]. Furthermore, a consistent clinical relationship exists between cytoplasmic HuR protein and patient survival. HuR affects the treatment responses of anti-cancer drugs by stabilization of specific mRNAs. Ultimately, these findings could prove helpful in identifying a therapeutic or prognostic target. However, an important challenge will be to elucidate the regulatory mechanism of HuR and its structural modifications in cancer, which will contribute to the validation of pharmaceutical strategies.

## Figures and Tables

**Table 1:** Exogenous regulators that influence the expression and function of HuR.

Regulators	Effect on HuR	References
UVR	Cytoplasmic accumulation ↑	[27,28]
Compound		
Ethanol	Cytoplasmic accumulation ↑	[29,30]
LPS	Cytoplasmic accumulation ↑	[31,32]
SAHA	Protein ↓	[44]
Tamoxifen	Cytoplasmic accumulation ↑	[33]
Gemcitabine	Cytoplasmic accumulation ↑	[34]
Nitric oxide	mRNA ↓, protein ↓	[23]
HIV protease inhibitor	Cytoplasmic accumulation ↑	[35]
Proteasome inhibitor MG132	Cytoplasmic accumulation ↑	[36]
Microenvironment change		
Hypoxia	Cytoplasmic accumulation ↑	[37]
Amino acid limitation	Cytoplasmic accumulation ↑	[38]
Bile salts	Cytoplasmic accumulation ↑	[39]
Serum	Cytoplasmic accumulation ↑	[40]
Polyamines depletion	Cytoplasmic accumulation ↑	[41–43]
DHA	Cytoplasmic accumulation ↑	[44]
Nature reagent		
Green tea	Cytoplasmic accumulation ↓	[45,70]
Ginkgo biloba extract	Cytoplasmic accumulation ↓	[71]
KPS-A	Cytoplasmic accumulation ↓	[47]
Triptolide	Cytoplasmic accumulation ↓	[48]
Cytokine		
IL-1β	Cytoplasmic accumulation ↑	[49]
TNF-α	Cytoplasmic accumulation ↑	[50]
TGF-β1	Cytoplasmic accumulation ↑	[51]
Virus infection		
HPV	Cytoplasmic accumulation ↑	[52,53]
Alphavirus	Cytoplasmic accumulation ↑	[54]
Hormone		
ACTH	Cytoplasmic accumulation ↑	[55]
Androgens	Cytoplasmic accumulation ↑	[56,57]
17β-estradiol	Cytoplasmic accumulation ↑	[58]

UVR, ultraviolet radiation; LPS, lipopolysaccharide; SAHA, suberoylanilide hydroxamic acid; DHA, docosahexaenoic acid; KPS-A, kalopanaxsaponin A; IL-1β, interleukin-1β; TNF-α, tumor necrosis factor-α; TGF-β1, transformation growth factor-β1; HPV, human papillomavirus; ACTH, adrenocorticotropic hormone.

**Table 2:** Endogenous regulators that influence the expression and function of HuR.

Regulators	Mechanism	Effect sites	Effect on HuR	References
NF-κB	Transcriptional	Promoter	HuR mRNA ↑	[72]
Smad	Transcriptional	Promoter	HuR mRNA ↑	[73]
Kinases				
PKCα	Phosphorylation	S158, S221	RNA-binding ↑, cytoplasmic accumulation ↑	[61]
PKCδ	Phosphorylation	S318, S221	RNA-binding ↑, cytoplasmic accumulation ↑	[62]
Cdk1	Phosphorylation	S202	Cytoplasmic accumulation ↑	[63]
Chk2	Phosphorylation	S88, S100, T118	RNA-binding ↑	[64]
p38	MAPK Phosphorylation	T118	Cytoplasmic accumulation	[65]
PI3K-AKT	Transcriptional.	Promoter	p65/RelA binding to a putative NF-κB binding site in the HuR promoter ↑	[72]
AMPK	Transcriptional	K22 and S105 of importin α	Nuclear import via phosphorylation and acetylation of importin α ↑	[41,68,69]
miRNAs				
miR-9	Transcriptional	Unknown	HuR mRNA↓; HuR protein ↓	[74]
miR-200c	Unknown	Unknown	Interaction of HuR and mRNA ↓	[75]
miR-9	Post-transcriptional	3′UTR	HuR mRNA↓; HuR protein ↓	[76]
miR-34a	Post-transcriptional	3′UTR	HuR mRNA↓; HuR protein ↓	[77]
miR-16	Translational	3′UTR	HuR protein ↓	[78]
miR-125a	Translational	3′UTR	HuR protein ↓	[79]
miR-519	Translational	3′UTR	HuR protein ↓	[80–82]
Proteins				
CARM1	Methylation	R217	RNA-binding ↑, cytoplasmic accumulation ↑	[66]
HuR	Ubiquitinylation	K182	Protein stability ↑	[67]
HuR	Post-transcriptional	polyadenylation site	mRNA stability ↑	[83]
Hu (B-D)	Post-transcriptional	polyadenylation site	mRNA stability ↑	[84]
TTP	Post-transcriptional	3′UTR	mRNA stability ↓	[83]
pp32	Interaction	Not indicated	RNA-binding ↓	[85]
RNP C1	Post-transcriptional	RRM, 3′UTR	RNA-binding ↑, mRNA stability ↓	[86,87]
Mdm2	Ubiquitinylation	K283, K313, K326	Protein stability ↑	[88]
Hsf1	Not indicated	Not indicated	HuR protein ↑	[89]

NF-κB, nuclear factor-κB; PKC, protein kinase; Cdk1, cyclin-dependent kinase 1; Chk2, checkpoint kinase 2; p38 MAPK, p38 mitogen-activated protein kinase; PI3K-AKT, phosphatidylinositol 3-kinase AKT; AMPK, AMP-activated protein kinase; miRNA, microRNA; CARMI, coactivator-associated arginine methyltransferase 1; TTP, tristetraprolin; pp32, protein phosphatase 32; Mdm2, murine double minute 2; Hsf1, heat shock transcription factor.

**Table 3:** The association between HuR expression and patient outcome in human cancer.

First author	Year	Country	Method	*N*	Prognostic effect of HuR		Type of cancer
						
					Unadjusted	Adjusted	
Miyata *et al.* [109]	2013	Japan	IHC	122	C b,f↓	C b,f↓	Bladder Cancer, pTa-3
Zhu *et al*. [167]	2013	China	IHC	82	C a,c↓, N a,c (NS)	C a,c↓, N a,c (NS)	Breast cancer, stage I–III
Lauriola *et al*. [150]	2012	Italy	IHC	54	C b,c↓, NCR b,c↓, N b,c (NS)	C b,d↓, NCR b,c	Lung adenocarcinoma, stage I–II
Kim *et al*. [98]	2012	South Korea	IHC	96	C c (NS), N c (NS)	C c (NS), N c (NS)	Oral squamous cell carcinoma, stage I–IV
Liang *et al*.[148]	2012	China	IHC	340	C a,b,f↓	C f (NS)	Upper urinary tract urothelial carcinoma
Kim *et al*. [106]	2011	South Korea	IHC	244	C c (NS), N c (NS), (C + N) c (NS), (C − N) c (NS)	C c (NS), N c (NS), (C + N) c (NS), (C − N) c (NS)	Lung adenocarcinoma and squamous cell carcinomas, I–IV
Yuan *et al*. [170]	2011	UK	RT-PCR	109	HuR mRNA a,c (NS)	HuR mRNA a,c (NS)	Invasive breast carcinoma, stage I–IV
Ronkainen *et al*. [165]	2011	Finland	IHC	152	C c↓	C c (NS)	Renal cell carcinoma, stage I–IV
Wang *et al*. [102]	2011	China	IHC	132	C a,c↓, N a,c (NS)	C a,d↓, N a,c (NS)	Non-small cell lung carcinoma, I–IIIB
Cha *et al*. [166]	2011	South Korea	IHC	103	C c↓, N a,c (NS)	C d↓, N a,c (NS)	Oral squamous cell carcinoma, I–IV
Richards *et al*. [142]	2010	USA	IHC	52	C d↑	C d↑	Pancreatic ductal adenocarcinoma
Mrena *et al*. [107]	2010	Finland	IHC	316	C c↓	C c (NS)	Gastric carcinoma, stage I–IV
Costantino *et al*. [34]	2009	USA	IHC	32	C d↑	C d↑	Pancreatic ductal adenocarcinoma
Yi *et al*. [168]	2009	USA	IHC	113	N a↓	N a↓	Ovarian carcinoma, stage I–IV
Yoo *et al*. [141]	2009	USA	Immunofluorescence	560	(C + N) c↓, NCR c↑	(C + N) c↓	Colorectal carcinoma, stage I–IV
Stoppoloni *et al*. [108]	2009	Italy	IHC	29	C c↓	Not indicated	Mesothelioma
Ortega *et al*. [169]	2008	Spain	Western blotting	89	(C + N) a↑	Not indicated	Invasive breast carcinoma
Niesporek *et al*. [138]	2008	Germany	IHC	104	C a (NS), N a↑	N a↑	Prostate carcinoma
Heinonen *et al*. [110]	2007	Finland	IHC	641	C c,e↓	C c↓	Invasive breast carcinoma
Lim *et al*. [112]	2007	South Korea	IHC	308	C c (NS), N c (NS)	C c (NS), N c (NS)	Cervical carcinoma, carcinoma in situ and stage I–II
Denkert *et al*. [99]	2006	Germany	IHC	87	C c (NS)	C c (NS)	Colorectal carcinoma,
Mrena *et al*. [100]	2005	Finland	IHC	316	C c↓	C c (NS)	Gastric carcinoma, stage I–IV
Heinonen *et al*. [111]	2005	USA	IHC	133	C a↓	C a↓	Invasive breast carcinoma, stage I–III
Denkert *et al*. [103]	2004	Germany	IHC	208	C a,c (NS), N a,c (NS)	C a,c (NS), N a,c (NS)	Invasive breast carcinoma, stage I–III
Denkert *et al*. [104]	2004	Germany	IHC	83	C a,c↓, N a,c (NS)	C a,c↓	Ovarian carcinoma, stage I–IV
Erkinheimo *et al*. [140]	2003	Finland	IHC	445	C c↓, N c (NS)	C c (NS)	Ovarian carcinoma, stage I–IV

IHC, immunohistochemistry; C, cytoplasmic HuR; *N*, nuclear HuR; NCR, nuclear-to-cytoplasmic ratio; C + N, total cytoplasmic and nuclear HuR; C − N, difference between cytoplasmic and nuclear HuR; NSCLC, non-small cell lung cancer; NS, not significant;

a disease-free survival;

b metastasis-free survival;

c overall survival;

d overall survival for patients treated with gemcitabine;

e in familial non-BRCA1/2 breast carcinoma;

f disease-specific survival.
